# Study on anti-frost heave effect of new thermal insulation subgrade of highway in seasonally frozen soil regions

**DOI:** 10.1371/journal.pone.0318682

**Published:** 2025-02-04

**Authors:** Xiaoqiang Mi, Wenqiang Zhang, Ge Zhang, Xinbin Wang

**Affiliations:** 1 Gansu Wantai Construction Engineering Company Limited., Lanzhou, China; 2 Northwest Institute of Eco-Environment and Resources, Key Laboratory of Cryospheric Science and Frozen Soil Engineering, Chinese Academy of Sciences, Lanzhou, China; 3 College of Civil Engineering & Architecture, China Three Gorges University, Yichang, China; Shandong University of Technology, CHINA

## Abstract

Frost heave is one of the important factors affecting the long-term stability and safe operation of subgrade in seasonally frozen soil regions. To investigate the anti-frost heave effect of foam concrete insulation and composite insulation structure subgrade, a multi-physics fields coupling numerical simulation was employed. From the perspective of hydrothermal process of the subgrade, the ground temperature distribution, maximum freezing depth, ice distribution, and maximum frost heave are systematically analyzed. The results indicate that the foamed concrete insulation layer subgrade exhibits a significant anti-frost heave effect. Meanwhile, to alleviate the sunny-shady slopes effect on subgrade in the seasonally frozen soils regions, the composite insulation structure combining XPS insulation board with foamed concrete insulation was recommended. Moreover, the appropriate anti-frost heave measures for subgrade are proposed, which considering the different frost heave characteristics. The findings can serve as a reference for the prevention and control of frost heave in highway subgrade and for the application of foam concrete insulation layers in seasonal frozen soil regions.

## 1. Introduction

Frozen soils region refers to the region where the soil temperature is below 0°C and contains ice crystals. According to the freeze duration, frozen soils is often divided into seasonal frozen soils and permafrost. Among them, the seasonal frozen soil areas accounts for 53.5% of China’s land areas and is widely distributed in Northwest, Northeast and North of China [1, 2]. The subgrade engineering in seasonal frozen soils areas often suffers from multiple freeze-thaw cycles. It leads to uneven frost heave, melt settling and other subgrade engineering diseases, which greatly limits the transportation capacity [3, 4]. With the intensification of global warming [5] and humidification [6], the problems of subgrade causing by frost heave in seasonal frozen soils areas have become increasingly prominent. Under the negative temperature, the pore water in the subgrade soils freezes into ice, resulting in the volume expansion of subgrade soils. At the same time, under the action of negative temperature gradient, the water in the lower unfrozen layer continuously migrates into frozen area. These lead to large expansion of subgrade soils, which can cause uneven deformation of subgrade [7]. It is necessary to carry out the research on the prevention and control technology of frost heave disease of highway subgrade in seasonal frozen soil area.

Frost heave disease in frozen soils engineering is the macroscopic manifestation of water-heat-force interaction in subgrade soils [8]. Researches show that moisture, frost heave sensitive soil and negative temperature are the three major factors [9–11]. Three factors must be met at the same time, controlling one of them can inhibit subgrade frost heave. Therefore, the insulation layer method is used to reduce the external cold energy transfer into the subgrade soils, which can alleviate the frost heave deformation of the subgrade [12, 13]. By coupling analysis of water field, temperature field and deformation field in the freeze-thaw process of subgrade soil, some theoretical guidance for the design and construction of subgrade engineering in frozen soils areas are provided [14]. The laboratory experiment results show that laying insulation board can effectively reduce the freezing depth of subgrade soils [15]. It found that the amount of frost heave decreases with the thickness of insulation board increasing, which shows good anti-frost heave effect. Cai et al. [16] conducted field tests and field monitoring for the materials, especially for thermal performance, elastic deformation, and accumulated deformation of insulation materials. Experiment results show that mechanical properties of full section insulation layer structure is stable. The finite element method was applied to discuss the best buried depth and location of thermal insulation board. The performance of thermal insulation board was explored by comparing the without thermal insulation board subgrade [17]. Xu et al. [18] made a comparative analysis of the influence of different measures on the maximum freezing depth of the subgrade by numerical analysis. The results show that the buried depth, buried location, thickness and material of the insulation board have important effects on the anti-frost heave effect of the subgrade. It is found that laying insulation board has a better anti-frost heave effect on the new high railway foundation.

Based on the principle of heat preservation to prevent frost heaving disease of subgrade in seasonal frozen soils areas, the foam concrete insulation layer which has high strength heat preservation, good durability and stability was applied [19]. Foam concrete is made of cement as the main gel material, which can be mixed with fly ash, fiber and other materials. It has the characteristics of high strength, earthquake resistance, sound absorption and heat preservation. Eva et al. [20] studied the durability of foamed concrete under cold wave environment. Chen et al. [21] study on the strength deterioration mechanism of foamed concrete under freeze-thaw cycles by: experiment and numerical simulation methods. The results show that during F-T cycles, the strength of FC exhibits a declining trend, which becomes increasingly pronounced with the rise in the number of F-T cycles. Aiming at the prevention and control of frost heave disease of railway subgrade in seasonal frozen soil area, Zhuo et al. [13] analyzed the performance of foamed concrete under different admixture conditions for the prevention and control of frost heave disease of railway subgrade in seasonal frozen soils areas. Finally, the best performance of foamed concrete was obtained. However, the existing studies have not deeply discussed the influence of the thickness of foamed concrete insulation layer on the freeze-heave of highway subgrade in the seasonal frozen soil areas. The effects of thermal insulation structure on frost heave deformation are relatively insufficient from the perspective of water-heat process of subgrade soils and frost heave deformation. As the result, the anti-frost heave performance of foamed concrete insulation layer is not clear. However, the existing methods for long-term performance prediction are very extensive, including experiments [22], theoretical modeling [23, 24], numerical simulation [25, 26], and artificial intelligence [27–29]. The cost of testing the new thermal insulation structure is more expensive. Therefore, the numerical simulation method will be a more cost-effective and reasonable method.

Therefore, applying the hydrothermal multi-field coupling calculation method to systematically analyze the influence of foam concrete insulation layer on the temperature field, ice content and deformation field of subgrade in the seasonal frozen soil areas. The influence of insulation layer thickness and composite structure (XPS insulation board + insulation layer on both sides of the slope) on the frost heave of subgrade are analyzed. Finally, according to the simulation results, the most effective anti-frost heave measures are proposed.

## 2. Hydrothermal multi-field coupling numerical simulation

### 2.1 Basic governing equations

#### 2.1.1 Soil water transfer equation

Frost heave occurs during the process of soil freezing. This is because pore water in the soil becomes ice, resulting the soils volume increases. On the other hand, driven by the temperature gradient and hydraulic gradient, the pore water in unfrozen soils areas migrates to the freezing zone. The accumulated water freezes into ice lenses, which plays a leading role in soil frost heave. The water transfer equation of saturated or unsaturated frozen soil considering the phase change of ice- water during soil freezing can be expressed as [30–32]:

$$\frac{\partial \theta_{u}}{\partial t}+\frac{\rho_{i}}{\rho_{w}}\frac{\partial \theta_{i}}{\partial t}=\nabla [D(\theta_{u})\nabla \theta_{u}-K(\theta_{u})]$$ (1)

where *θ*_*u*_ and *θ*_*i*_ are the volume unfrozen water content and volume ice content respectively, *ρ*_*i*_ and *ρ*_*w*_ are the density of water and ice respectively, ∇ is a differential operator, *D*(*θ*_*u*_) is the water diffusion coefficient of soils, *K*(*θ*_*u*_) is the permeability coefficient of soils, *t* is the time.

Under negative temperature conditions, the unfrozen water of frozen soils is in the dynamic equilibrium state with negative temperature, pressure and other conditions. When the external pressure is constant, the content of unfrozen water can be expressed as the single value function of negative temperature, which is commonly expressed as an exponential function [33, 34]. It can be expressed as:

$$\theta_{u}=a_{1}{\left|T\right|}^{{-b}_{1}}$$ (2)

where *a*_1_ and *b*_1_ are test parameters related to soils properties, respectively.

It is worth noting that the formation of pore ice in the freezing process of soil can hinder the migration channel of water, which can reduce the permeability coefficient and water diffusion coefficient of frozen soils. Therefore, the impedance factor(*I*) is introduced to reflect the influence of pore ice on water migration during the freezing process. The permeability coefficient *K*(*θ*_*u*_) and diffusion coefficient *D*(*θ*_*u*_) soils can be expressed as [35, 36]:

$$K(\theta_{u})=\frac{a_{2}\theta_{u}^{b_{2}}}{I}=\frac{a_{2}\theta_{u}^{b_{2}}}{10^{10\theta_{i}}}$$ (3)

$$D(\theta_{u})=\frac{a_{3}\theta_{u}^{b_{3}}}{I}=\frac{a_{3}\theta_{u}^{b_{3}}}{10^{10\theta_{i}}}$$ (4)

where, *a*_2_, *b*_2_, *a*_3_ and *b*_3_ are physical parameters related to subgrade soils material.

#### 2.1.2 Heat conduction equation

During the heat transfer process of subgrade, ignoring the influence of convective heat transfer and only considering the influence of ice-water phase transition, the heat conduction equation can be expressed as [37]:

$$\rho c\frac{\partial T}{\partial t}=\lambda \nabla^{2}T+L\rho_{i}\frac{\partial \theta_{i}}{\partial t}$$ (5)

where ρ is the density of soils, c is the specific heat capacity of soils, T is the temperature; *λ* is the thermal conductivity of soils, *L* is the latent heat of the phase transition of water, which is 334.5 kJ·kg^-1^.

The migration and redistribution of water are related with temperature field in soils. The water field changes the thermal physical characteristics of soils. Conversely, the change of temperature field directly affects the migration and redistribution of water in soils. Especially, when the soils temperature is below the temperature of ice-water phase transition, the appearance of pore ice and the release of latent heat of phase transition can cause drastic changes of basic physical and thermophysical parameters of soils. Therefore, considering the interaction between temperature field and water field during frozen soils freezing, Eq (1) was substituted into Eq (5) to establish the hydrothermal coupling equation under freeze-thaw conditions [38]:

$$C^{*}\frac{\partial T}{\partial t}=\lambda^{*}\nabla^{2}T+L\rho_{w}\frac{\partial K(\theta_{u})}{\partial y}$$ (6)

where C* and λ* are equivalent volumetric heat capacity and equivalent thermal conductivity, respectively.

In which, C* can be expressed as:

$$C^{*}=c\rho +L\rho_{w}\frac{\partial \theta_{u}}{\partial T}$$ (7)

And, λ* can be expressed as:

$$\lambda^{*}=\lambda +L\rho_{w}D(\theta_{u})\frac{\partial \theta_{u}}{\partial T}$$ (8)

#### 2.1.3 Stress-strain equation

1. *Differential equation of soil equilibrium.* In soil mechanics, it is generally assumed that soil pressure is positive. The equilibrium differential equation can be expressed as [33]:
$${\left[\partial \right]}^{T}\{\sigma \}-\{F\}=0$$ (9)
  where [∂] is the differential operator, can be expressed as $[\partial ]={\left[\begin{array}{ccc} \frac{\partial }{\partial x} & 0 & \frac{\partial }{\partial y}\\ 0 & \frac{\partial }{\partial x} & \frac{\partial }{\partial y} \end{array}\right]}^{T}$, {*σ*} is the stress tensor, {*F*} is the soil volume force, $\{F\}={\left\{\begin{array}{cc} F_{x} & F_{y} \end{array}\right\}}^{T}$.
2. *Deformation equation*. Under the assumption of small deformation, the geometric equation of soil mass can be expressed as [39]:
{ε}=−[∂]{u} (10)
  where {ε} is strain tensor, $\{\varepsilon \}={\left\{\begin{array}{ccc} \varepsilon_{x} & \varepsilon_{y} & \tau_{xy} \end{array}\right\}}^{T}$, {*u*} is the displacement, $\{u\}={\left\{\begin{array}{cc} u_{x} & u_{y} \end{array}\right\}}^{T}$.
3. *Constitutive equation*. Under the action of external load, the constitutive relation of soils can be expressed as [39]:
$$\left\{\Delta \sigma \}=[D_{T}](\{\Delta \varepsilon \}-\{\Delta \varepsilon_{v}\}-\{\Delta \varepsilon_{vp}\}\right)$$ (11)
  where {Δσ} is stress increment, [*D*_*T*_] is the temperature-dependent elastic stiffness matrix, can be expressed as $[D_{T}]=\frac{E_{T}(1-v_{T})}{\left(1+v_{T})(1-2v_{T}\right)}\left[\begin{array}{ccc} 1 & \frac{v_{T}}{1-v_{T}} & 0\\ \frac{v_{T}}{1-v_{T}} & 1 & 0\\ 0 & 0 & \frac{1-{2v}_{T}}{2(1-v_{T})} \end{array}\right]$. And the *E*_*T*_ and *v*_*T*_ are the temperature-dependent elastic modulus and Poisson’s ratio, respectively. {Δ*ε*}, {Δ*ε*_*v*_} and {Δ*ε*_*vp*_} are total strain increment, frost heave strain increment and plastic strain increment, respectively.

In the process of soil freezing, soil frost heave is mainly caused by the in-situ freezing of water and the increasing migration water freezing into ice. The deformation caused by soil frost heave can be expressed as [40]:

$$\varepsilon^{v}=\left\{ \begin{array}{cc} \theta_{i}+\theta_{u}-n_{0} & \theta \geq \theta_{s}\\ \beta (\theta_{i}+\theta_{u}-\theta_{0}) & \theta <\theta_{s} \end{array}\right.$$ (12)

where *β* is the effective strain ratio, *n*_0_ is the initial porosity of soils, *θ*_0_ is the initial moisture content, *θ*_*s*_ is the saturated moisture content. Under the plane strain state, the soil strain increment is as follows:

$$\{\varepsilon_{v}\}=\frac{\varepsilon^{v}}{3}{\left\{\begin{array}{ccc} 1+v_{T} & 1+v_{T} & 0 \end{array}\right\}}^{T}$$ (13)

Under complex stress conditions, the plastic strain increment of soils can be expressed as [33],

$${d\varepsilon }_{vp}=d\lambda \frac{\partial Q}{\partial \{\sigma \}}$$ (14)

where *d*λ is plastic increment multiplier, *Q* is the plastic potential function.

This paper uses the Drucker-Prager yield criterion associated with the Mohr-Comlomb criterion:

$$\frac{\partial Q}{\partial \{\sigma \}}=\frac{\partial F}{\partial \{\sigma \}}={\left[\begin{array}{ccc} \frac{\partial F}{\partial \sigma_{x}} & \frac{\partial F}{\partial \sigma_{y}} & \frac{\partial F}{\partial \tau_{xy}} \end{array}\right]}^{T}$$ (15)

where: *F* is the yield function.

The coupled moisture-heat equation in Eqs (1)—(15) constitute the coupled moisture-heat-deformation model of subgrade. This paper uses the COMSOL Multiphysics numerical software to solve the model.

### 2.2 Calculation model and related parameters

**Fig 1:**
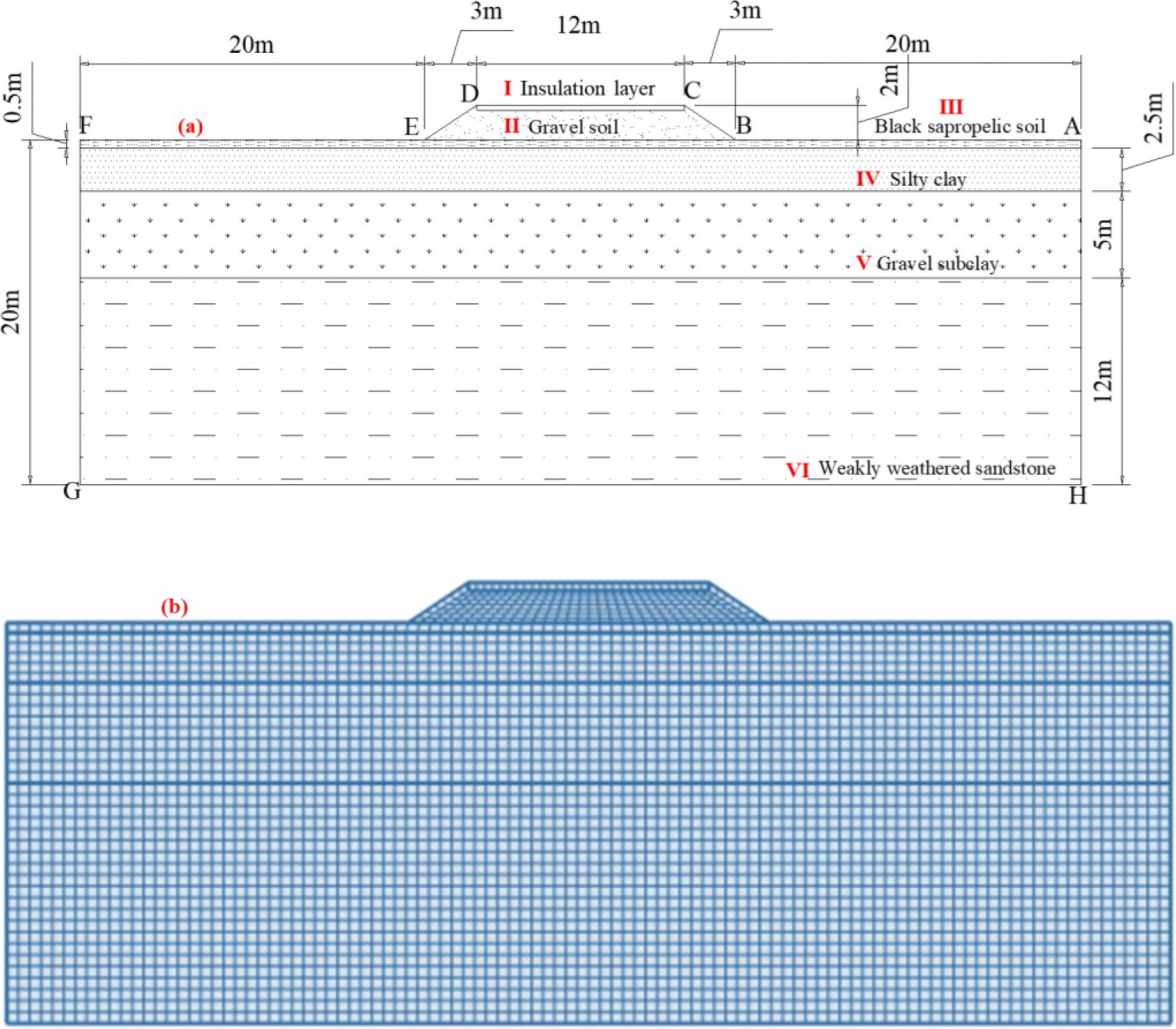
Calculated model. (a) Physical structures; (b) Finite element model.

Taking the subgrade in the mountainous area of southern Gansu Province in the seasonal frozen soil as an example [41], the calculation model of subgrade anti-frost heave measures is established according to the engineering geological conditions and geotechnical properties of the test site (Fig 1). the subgrade width (DC) is 12m, the embankment height is 2m, and the slope of subgrade is 1:1.5. The extension width (EF) of both sides of subgrade is 20m. and the depth is 20m below the natural ground. The strata are as follows: gravel soil (II), black sapropelic soil (III), silty clay (IV), gravel subclay (V), weakly weathered sandstone (VI). To study the anti-frost heave effect of the new subgrade insulation material and optimize the structural parameters, the thickness and buried position of the insulation layer (I) in the subgrade was adjusted. The height and size of other strata do not change.

According to the existing test results, the thermal physical parameters and hydraulic parameters of each soil layer are shown in **Tables 1** and **2** [3, 21, 33].

**Table 1:** Thermophysical parameters of each soil layer of subgrade.

Soil layer	λ_f_ (W/m·°C)	λ_u_ (W/m·°C)	C_f_ (J/kg·°C)	C_u_ (J/kg·°C)	Ρ (kg/m^3^)
I	0.08	0.08	1550	1550	550
II	1.92	1.68	1140	1246	2070
III	1.60	1.26	1310	1750	1510
Ⅳ	1.72	1.31	1192	1369	1710
Ⅴ	1.75	1.45	1183	1308	1750
Ⅵ	1.82	1.68	1153	1258	1800

**Table 2:** Seepage parameters of each soil layer of subgrade.

Soil layer	a_1_	b_1_	a_2_ (m/s)	b_2_	a_3_ (m^2^/s)	b_3_	θ_0_ (%)
I	-	-	-	-	-	-	0
II	0.34	-0.20	1.5×10^−5^	12.19	1.5×10^−6^	4.05	20
III	0.48	-0.16	1.4×10^−5^	11.59	1.4×10^−6^	3.95	28
Ⅳ	0.24	-0.15	1.34×10^−5^	14.59	1.3×10^−6^	4.18	16
Ⅴ	0.46	-0.15	4.7×10^−5^	11.27	2.0×10^−6^	7.18	12
Ⅵ	0.58	-0.05	2.55×10^−5^	5.57	2.17×10^−6^	1.95	12

Frozen soils is temperature sensitive material. According to the mechanics of frozen soils, mechanical parameters of frozen soils are closely related to temperature, including elastic modulus, Poisson’s ratio, cohesion and internal friction angle, etc., which can be expressed as [33]:

$$E_{T}=\left\{ \begin{array}{cc} a_{4}+b_{4}{\left|T\right|}^{0.6} & T\leq 0\\ a_{4} & T>0 \end{array}\right.$$ (16)

$$v_{T}=\left\{ \begin{array}{cc} a_{5}+b_{5}|T| & T\leq 0\\ a_{5} & T>0 \end{array}\right.$$ (17)

$$c=\left\{ \begin{array}{cc} a_{6}+b_{6}{\left|T\right|}^{c_{6}} & T\leq 0\\ a_{6} & T>0 \end{array}\right.$$ (18)

$$\varphi =\left\{ \begin{array}{cc} a_{7}+b_{7}{\left|T\right|}^{c_{7}} & T\leq 0\\ a_{7} & T>0 \end{array}\right.$$ (19)

where *a*_*i*_(4~7), *b*_*i*_(4~7) and *c*_*i*_(4~7) are the test parameters, respectively.

The mechanical parameters of each soil layer are shown in **Table 3**.

**Table 3:** Mechanical parameters of each soil layer of subgrade.

Soil layer	a_4_ /MPa	b_5_	a_5_	b_5_	a_6_ /kPa	b_6_	c_6_	a_7_ /°	b_7_	c7
I	200	0	0.3	0	-	-	-	-	-	-
II	24.7	12.3	0.32	-0.008	27.8	7	1.24	20	1.15	1.1
III	22.5	11.5	0.30	-0.008	26.5	6.7	1.22	19	1.05	1.1
Ⅳ	24.1	12.1	0.29	-0.008	25.4	6.3	1.12	19	0.95	1.1
Ⅴ	61	53	0.35	-0.007	10	37	1.0	15	0.75	1.1
Ⅵ	140	108	0.25	-0.004	100	24	1.1	25	0.9	1.2

### 2.3 Boundary conditions

According to the field surface temperature monitoring data [41], the temperature conditions of the upper surface of the calculated region can be simplified into trigonometric function [42]. For the seasonal frozen soil areas, climate warming can properly slow down the development of subgrade soils frost heave. For the northwest area, the solar radiation is high and the subgrade is east-west. Due to the difference of solar radiation on the subgrade slope, it is easy to produce significant shady-sunny slope effect. Therefore, the influence of the shady-sunny slopes effect is considered in the numerical calculation. Based on this, the temperature boundary conditions are shown as follows:

1. The temperature of EF and IJ sides of the natural surface varies according to the following formula:
$$T=6+12sin(\frac{2\pi t}{365}+\frac{\pi }{2})$$ (20)
2. The temperature of the top surface (AB) of the subgrade changes according to the sine law:
$$T=7+19sin(\frac{2\pi t}{365}+\frac{\pi }{2})$$ (21)
3. The temperature on the shady slope (BCDE) of the subgrade can be expressed as:
$$T=1+15sin(\frac{2\pi t}{365}+\frac{\pi }{2})$$ (22)
4. The temperature on the sunny slope (ALKJ) of the subgrade can be expressed as:
$$T=7+18sin(\frac{2\pi t}{365}+\frac{\pi }{2})$$ (23)
5. The distance between the edge FG(AH) and the slope foot of the subgrade is 20m. The thermal effect on the subgrade can be considered to be negligible, which can be regarded as an adiabatic boundary. The HG edge can be considered in terms of heat flux 0.06W /m^2^.
6. The focus of this paper is to study subgrade freezing characteristics under different anti-frost heave measures. Therefore, the influence of precipitation and water evaporation is not considered in the calculation. All water boundary is assumed to be non-permeable boundary. In addition, the FG and AH edges fixed the horizontal displacement. The bottom boundary HG fixed vertical displacement. The top boundaries IJ, KL, AB, CB and EF are displacement free boundaries.
7. Firstly, the temperature of each soil layer in the natural site is assumed to be the definite average value. The initial water content is assigned to each soil layer. The initial displacement of each soil layer in the natural site is assumed to be zero. The gravity is considered in the calculation process. Secondly, the natural boundary conditions are used to calculate the initial low temperature of the natural site. When the difference of annual average ground temperature of natural site is less than 0.01°C, the temperature field is considered to be stable. At this time, the temperature of each point is selected as the initial temperature field of each soil layer of natural site. Finally, the temperature field of the site after the subgrade construction is calculated. Assuming that the subgrade construction is completed at the time of the highest temperature in a year (that is, July 15), the phase angle is set to 0. At this time, the initial temperature of the subgrade is set as the highest temperature of the natural surface in a year.

### 2.4 Model verification

**Fig 2:**
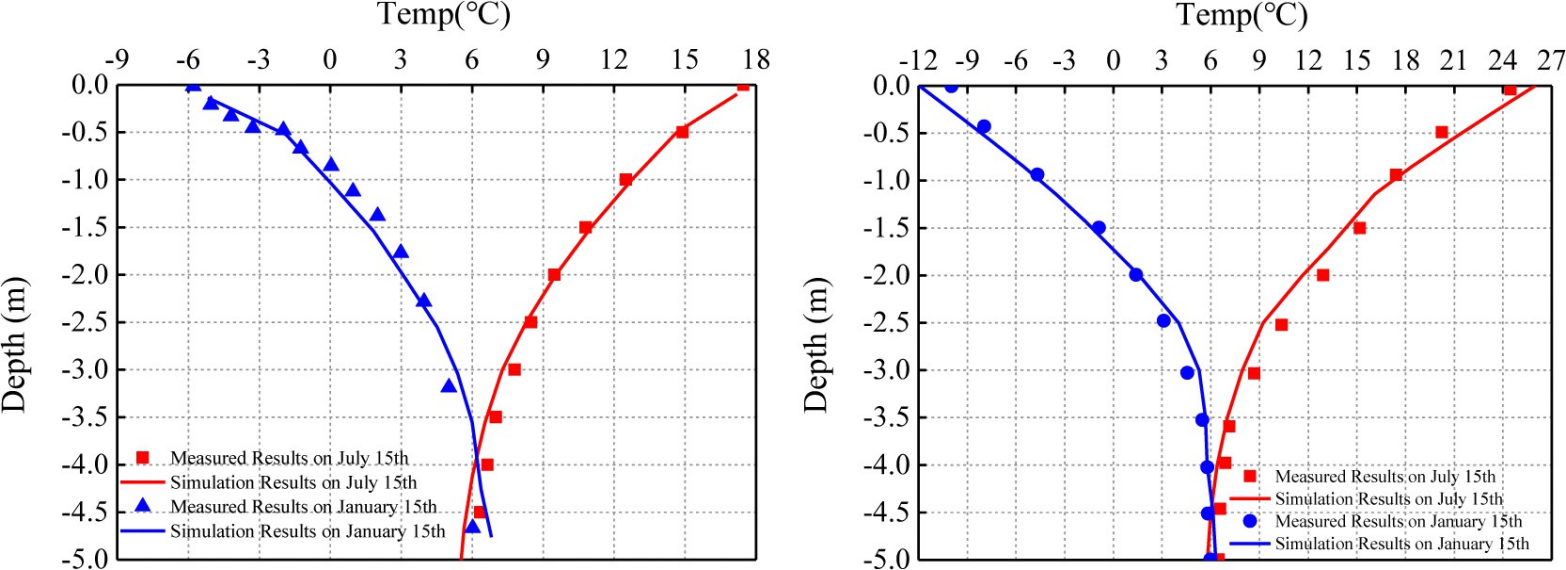
Comparison between measured and simulated temperature: (a) natural site; (b) subgrade central.

To verify the rationality and accuracy of the proposed model, the calculated results of ground temperature of natural site and subgrade were compared with the monitoring results [41]. The **Fig 2** shows the temperature simulation results of the natural field near the subgrade in the warm season (July 15) and cold season (January 15) are in good agreement with the monitoring values. However, in both cold and warm seasons, there are some differences between the calculated temperature and the monitoring results, but the error value is small. This phenomenon is caused by the fact that the boundary conditions of the model are ideal. The surface boundary is greatly affected by the land surface process, which is easy to cause calculation errors. At the same time, the parameters of each soil layer in the numerical simulation process are simplified, which can affect the numerical simulation results. The calculation and monitoring results of ground temperature at different time in the center of the subgrade after construction are compared and analyzed. Although there is a certain error between the measured results and the calculated results near the depth of -2.5m. The simulated curves almost coincide with the measured curves in other depth ranges. It can be proved that the above theoretical model can reasonably reflect the temperature field in the subgrade.

## 3. Analysis of anti-frost heave effect of foamed concrete

To investigate the influence of foamed concrete insulation layer thickness on the anti-frost heave effect of subgrade, the variation laws of ground temperature, maximum freezing depth, volume ice content and maximum frost heave of subgrade with different thickness of foamed concrete insulation layer are analyzed. The optimum thickness of subgrade with foamed concrete insulation layer is given. In view of the Shady—Sunny slopes effect of subgrade in seasonal frozen soils areas, the composite insulation structure combining XPS insulation board and foamed concrete insulation layer is proposed. The variation laws of ground temperature, maximum freezing depth and maximum frost heave of subgrade with different insulation thickness are simulated and analyzed.

### 3.1 Analysis of anti-frost heave effect with different thickness of foamed concrete

**Fig 3:**
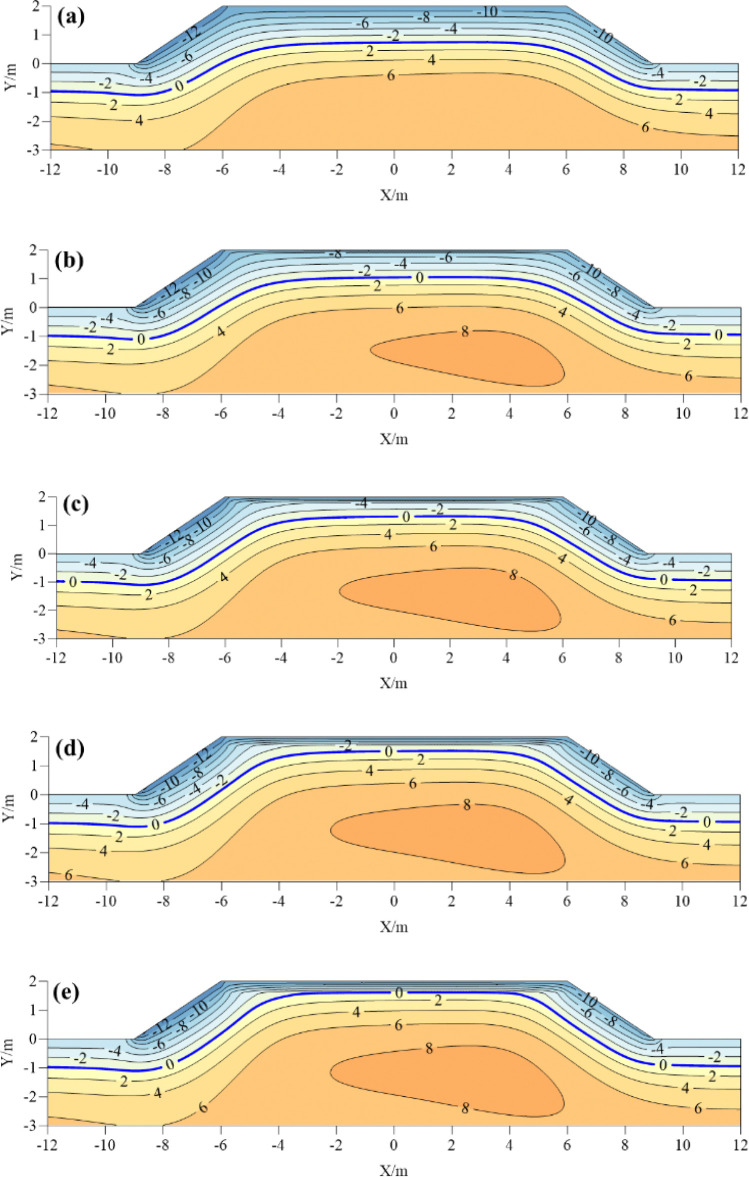
Ground temperature distribution map of subgrade with different insulation thickness at the coldest moment: (a) common roadbed; (b) 0.1 m; (c) 0.2m; (d) 0.3m; (e) 0.4m.

Fig 3 shows the ground temperature distribution of common subgrade and foamed concrete insulation subgrade with different insulation thickness (0.1m, 0.2m, 0.3m and 0.4m) at the coldest moment (January 15). The shady slope shoulder, the center of the subgrade and the sunny slope shoulder of subgrade are selected as the typical analysis points. From Fig 3(A) to Fig 3(E), it can be seen that the freezing depths of the shady slope shoulder, the center of the subgrade and the sunny slope shoulder of the common subgrade at the coldest moment are 1.65m, 1.41m and 1.50m, respectively. When the foamed concrete insulation layer thickness is 0.1m, the freezing depth of the shady slope shoulder, the center of the subgrade and the sunny slope shoulder are 1.45m, 0.97m and 1.15m, respectively. When the foamed concrete insulation layer thickness is 0.2m, the freezing depth of the shady slope shoulder, the center of the subgrade and the sunny slope shoulder are 1.30m, 0.69m and 0.85m, respectively. When the foamed concrete insulation layer thickness is 0.3m, the freezing depth of the shady slope shoulder, the center of the subgrade and the sunny slope shoulder are 1.18m, 0.51m and 0.85m, respectively. When the foamed concrete insulation layer thickness is 0.4m, the freezing depth of the shady slope shoulder, the center of the subgrade and the sunny slope shoulder are 1.06m, 0.39m and 0.84m, respectively. The results show that, the foamed concrete insulation layer can reduce the freezing depth of roadbed, compared with ordinary subgrade. With the increase of the thickness of the foamed concrete insulation layer, the freezing depth of the subgrade at different positions decreases gradually. When the thickness of the foamed concrete insulation layer increases from 0.1m to 0.4m, the freezing depth of the shady slope shoulder, the subgrade center and the sunny slope shoulder decrease by 0.39m, 0.58m and 0.31m, respectively. It can be proved that the laying of foamed concrete insulation layer has the most significant effect on the temperature at the center of the subgrade. The above results show that with the increase of thickness, the thermal resistance of cold flow into the subgrade increases, the cooling rate of the subgrade decreases, and the freezing depth of the subgrade gradually decreases. In addition, with the increase of the thickness of the foamed concrete insulation layer, the 8°C isotherm envelope area gradually increases, which also proves that the thickness of the insulation layer has a significant influence on the temperature changes of the subgrade.

**Fig 4:**
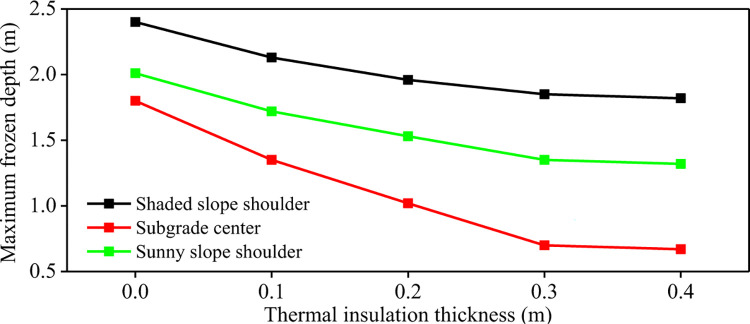
Maximum freezing depth of subgrade with different foam concrete insulation thickness.

After the ground temperature changes from positive temperature to negative temperature, the freezing line moves from shallow to deep, and the freezing depth gradually increases. When the freezing line reaches the maximum depth, the vertical distance between the ground and the maximum freezing line is called the maximum freezing depth. **Fig 4** shows the maximum freezing depth of the subgrade with different insulation thickness. The insulation thickness of 0(m) represents the common subgrade. With the increase of insulation layer thickness, the maximum freezing depth of different parts of the subgrade decreases gradually. When the thickness of foamed concrete insulation layer increases from 0m to 0.4m, the maximum freezing depth of subgrade center decreases by 62.78%. The maximum freezing depth of the shady slope shoulder and the sunny slope shoulder decreased by 24.17% and 34.33% respectively. When the thickness of the insulation layer exceeds 0.3m, the increase of the insulation layer thickness has no significant effect on the reduction of the maximum freezing depth of the subgrade, which is only reduced by 4.3%. Therefore, from the perspective of reducing the maximum freezing depth of subgrade, the thickness of the insulation layer set at 0.3m has the most significant effect on the frost heaving of the subgrade.

**Fig 5:**
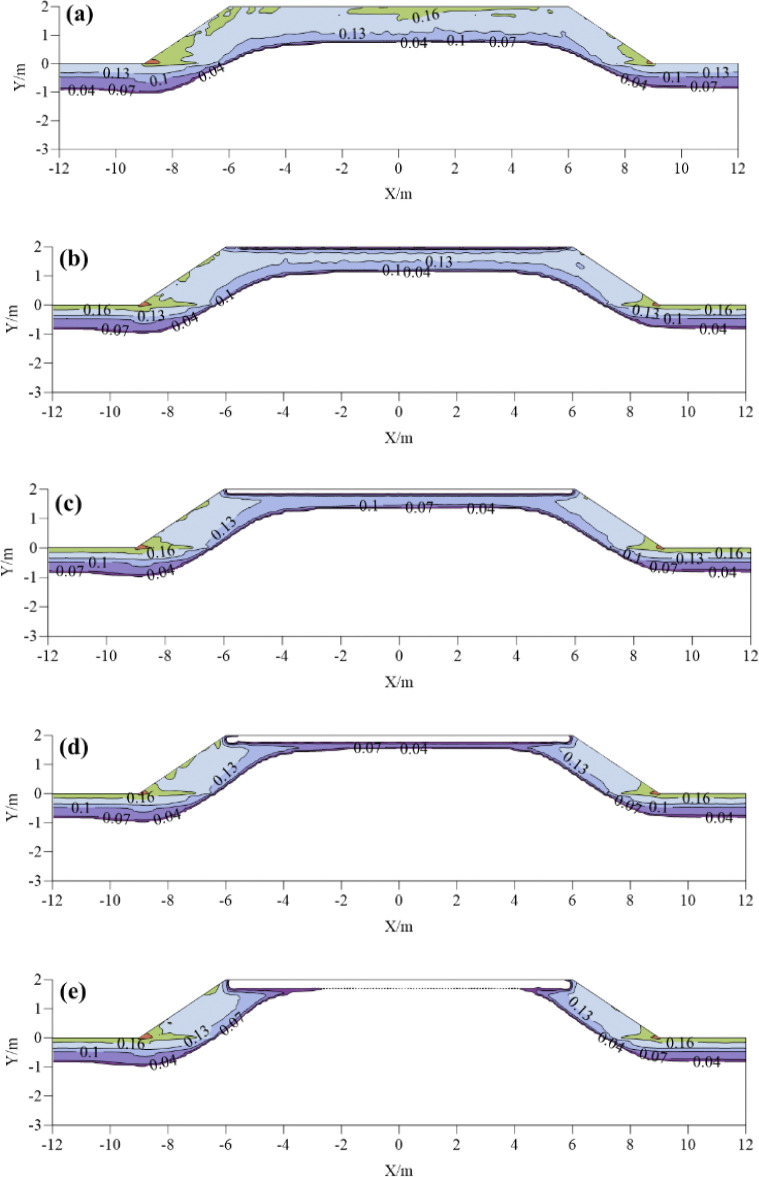
Distribution of ice content of subgrade with different insulation thickness at the coldest moment:(a) ordinary subgrade; (b) 0.1 m; (c) 0.2m; (d) 0.3m; (e) 0.4m.

The ice content of subgrade is the decisive factor for frost heave deformation of subgrade. When the volume of ice content is larger, the subgrade is more prone to frost heave failure. **Fig 5** shows the distribution of volume ice content of common subgrade and foamed concrete insulation layer subgrade with different thickness at the moment of cold season (January 15). For common subgrade, when the ambient temperature decreases, the ice content in the center of the roadbed can reach up to 17%. There is a certain amount of ice distributed on both sides of the subgrade. The shoulder of shady slope is more concentrated than that of the sunny slope. With the increase of the thickness of the foamed concrete insulation layer, the maximum volume ice content at the center of the subgrade decreases from 13% to 4%, indicating that the insulation layer significantly reduces the ice distribution area. With the increase of the thickness of insulation layer, not only the thermal resistance of cold flow into the subgrade is increased, but also the cooling rate of the subgrade is reduced. This reduces the amount of ice in the subgrade. In addition, it can be seen that when the thickness of the insulation layer is greater than 0.2m, the ice distribution areas and content in the lower part of the subgrade decrease significantly, which can effectively alleviate the frost heave of the subgrade.

**Fig 6:**
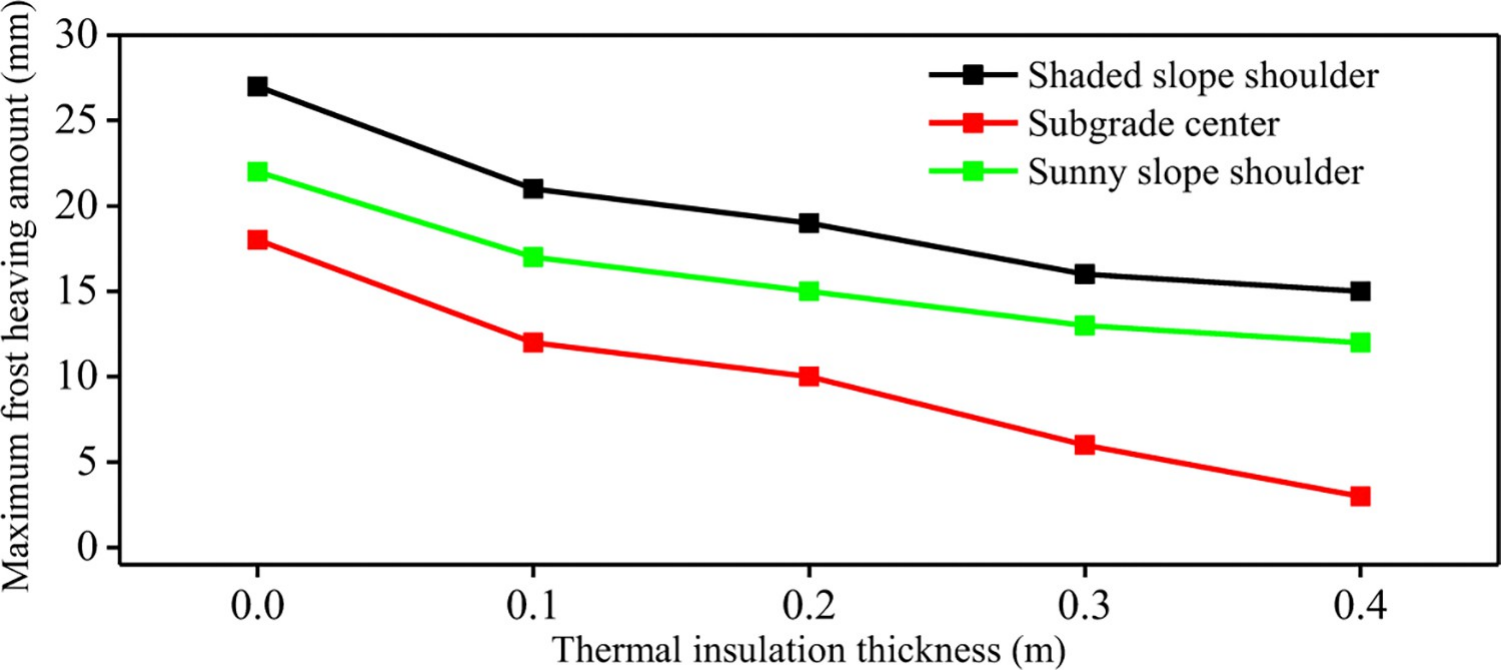
Maximum frost heave of subgrade with different insulation thickness.

**Fig 6** shows the maximum frost heave of the subgrade with different thickness of foamed concrete insulation layer. The results show that with the increase of the thickness of the foamed concrete insulation layer, the maximum frost heave amount in all parts of the subgrade decreases. The maximum frost heave amount in the center of the subgrade decreases most significantly. According to the principle of heat conduction, the thermal conductivity of insulation materials is different from that of soils. This causes a large temperature difference between the upper and lower parts of the insulation layer, resulting in thermal resistance effect. It lead the cold energy from above and below parts of the insulation layer is relatively small, and the maximum frost heave amount generated is small. It can also be seen that the thickness of the insulation layer can limit or delay the development of frost heave, the development of freezing depth and the increase of frost heave. But, the overall heat budget of the subgrade has not completely changed. The insulation layer has little influence on the frost heave quantity of the shady shoulder slope and the sunny slope shoulder, resulting in large frost heave quantity of the subgrade shoulder on both sides, which has a great influence on the long-term stability of the subgrade. Therefore, the new anti-frost heave measures should be sought to reduce the frost heave quantity on the shady slope and the sunny slope of the subgrade.

### 3.2 Analysis of anti-frost heave effect of composite insulation structure

To reduce the frost heave development of the subgrade shoulders on the shady slope and sunny slopes of the subgrade, a composite insulation structure combining 8cm thick XPS insulation board (on the shady slope and sunny slopes) and foamed concrete insulation layer (on the center of the subgrade) is proposed. Numerical simulation method is used to analyze the variation characteristics of the temperature field and maximum frost heave of the subgrade in the seasonal frozen soils areas under the composite insulation structure. The effect and applicability of the composite insulation structure are discussed.

**Fig 7:**
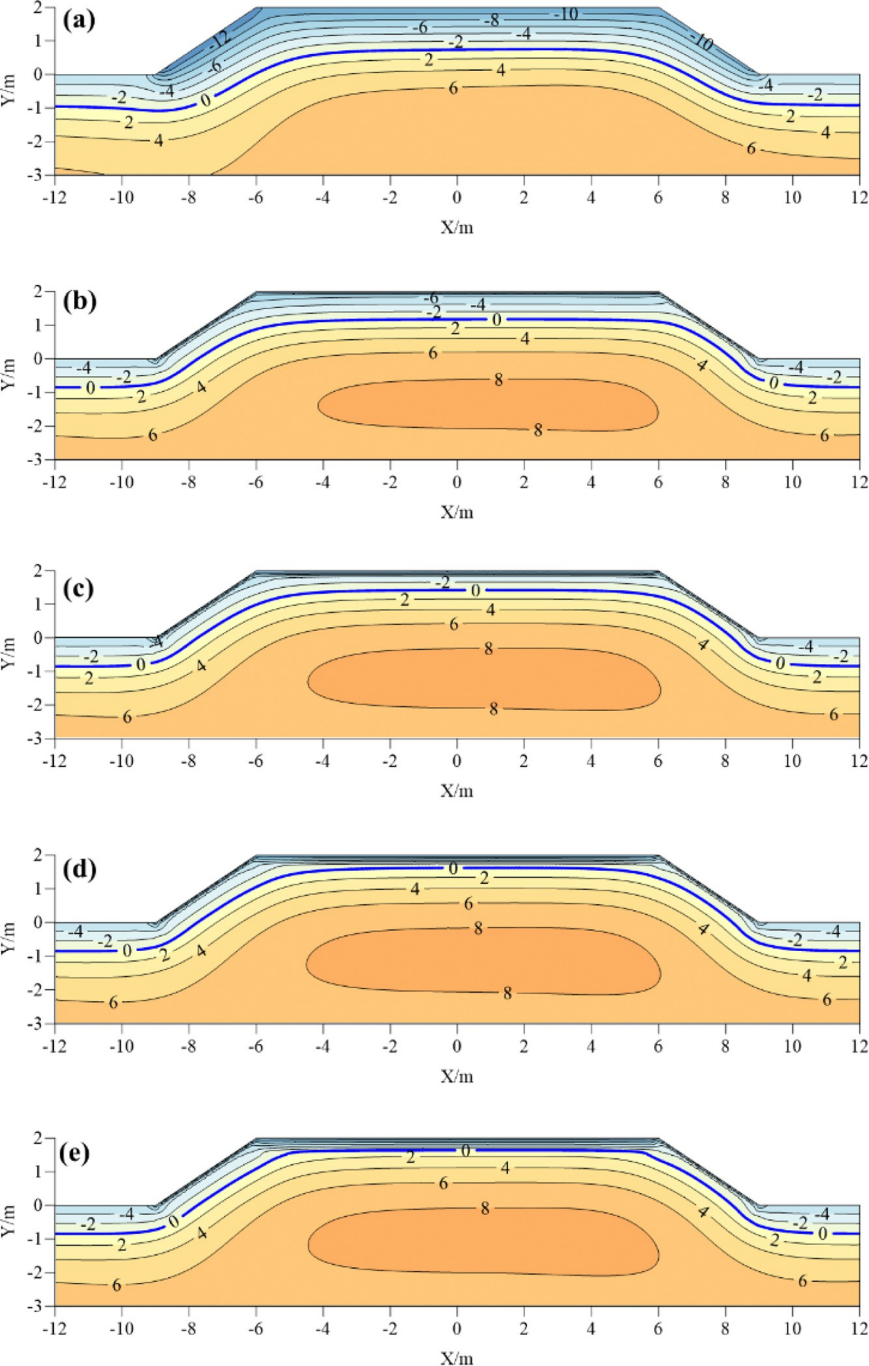
Temperature distribution of composite subgrade with different insulation thickness at the coldest moment: (a) common roadbed; (b) 0.1 m; (c) 0.2m; (d) 0.3m; (e) 0.4m.

**Fig 7** shows the temperature distribution diagram of the coldest moment of the composite insulation structure with different thickness of foamed concrete. Comparing the composite insulation structure with the common subgrade, it is found that the average temperature with the composite insulation structure is higher than that of the common subgrade. The zero temperature line of the composite insulation structure subgrade is raised. The temperature on both sides of the subgrade shoulder increases, and the sunny-shady slopes effect of subgrade is significantly weakened. With the increase of the thickness of the foamed concrete insulation layer at the center of the subgrade, the isothermal envelope area with the maximum temperature of 8°C gradually increases. Those indicate that the composite insulation structure blocks the external cold energy into the subgrade in the cold season, weakens the sunny-shady slopes effect of the subgrade, maintains the heat balance of the subgrade in a certain period of time, and maintains the stability of the subgrade.

**Fig 8:**
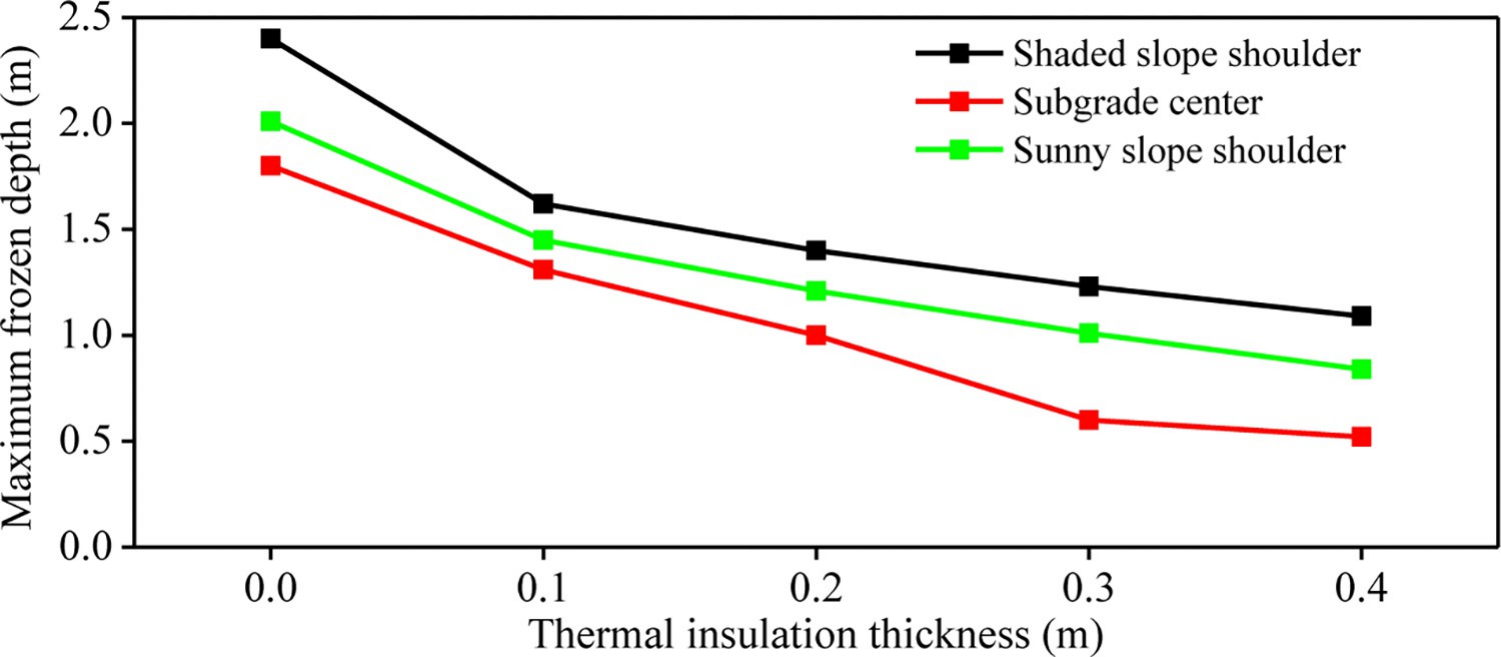
Maximum freezing depth of insulation structure subgrade with different foamed concrete thickness.

**Fig 8** presents the maximum freezing depth of composite insulation structure subgrade under different foamed concrete thickness. It is found that the maximum freezing depth of the subgrade center, the shady slope shoulder and the sunny slope shoulder are significantly reduced after lashady XPS insulation boards. The sunny-shady slopes effect is weakened. For common subgrade, the maximum freezing depth of subgrade center, shady slope shoulder and sunny slope shoulder are 1.80m, 2.40m and 2.01m, respectively. When the thickness of foamed concrete in the composite insulation structure is 0.1m, the maximum freezing depth of the subgrade center, shady slope shoulder and sunny slope shoulder are reduced to 1.31m, 1.62m and 1.45m respectively. The maximum freezing depth difference between the shady slope shoulder and the sunny slope shoulder of common subgrade is 0.39m. With the increase of insulation layer thickness, the maximum freezing depth of subgrade decreases gradually, and the maximum freezing depth of subgrade shoulder on shady slope and sunny slope decreases significantly. When the thickness of the foamed concrete is equal to or greater than 0.3m, the maximum freezing depth of the shady slope shoulder and the sunny slope shoulder with the XPS insulation board is reduced by more than 50% compared with the common subgrade.

**Fig 9:**
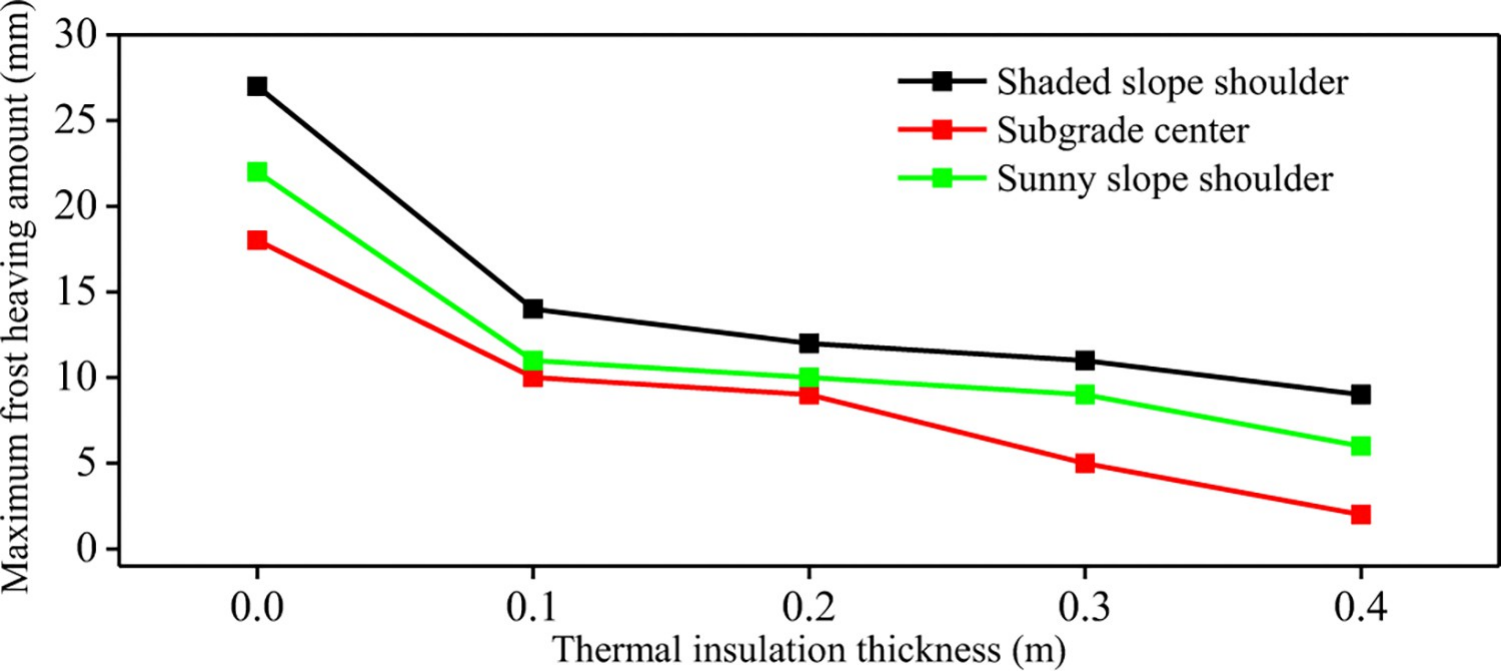
Maximum frost heaving amount of composite subgrade with different insulation thickness.

**Fig 9** shows the maximum frost heave curve of the composite insulation structure subgrade under different foam concrete thicknesses. It can be seen that the composite insulation structure significantly reduces the maximum frost heaving amount of the subgrade. For common subgrade, the maximum frost heaving amount of subgrade center, shady slope shoulder and sunny slope shoulder is 18mm, 27mm and 22mm, respectively. When the thickness of foamed concrete in the composite insulation structure is 0.1m, the maximum frost heaving amount of subgrade center, shady slope shoulder and sunny slope shoulder is 10mm, 14mm and 11mm, respectively. The frost heaving amount of monitoring points decreased by 44.4%, 48.1% and 50%, respectively. When the thickness of the foamed concrete of the composite structure increases from 0.1m to 0.4m, the maximum frost heaving amount of the subgrade center, shady slope shoulder and sunny slope shoulder decrease by 80%, 35.7% and 45.5% respectively.

### 3.3 Comparison of anti-frost heave effect of foam concrete insulation layer and composite insulation structure subgrade

From the above numerical simulation results, it can be seen that the foamed concrete insulation layer and the composite structure insulation layer have a certain effect on alleviating the frost heave deformation of subgrade. **Tables 4** and **5** give the maximum freezing depth and maximum frost heave amount of the foamed concrete insulation layer subgrade with different thickness and composite structure insulation layer with different thickness respectively. It is found that the anti-frost heave effect of the composite structure insulation layer subgrade is better than that of the foamed concrete insulation layer. Under the condition of the same thickness insulation layer, the maximum freezing depth and maximum frost heave amount of the composite structure insulation layer are smaller. For foamed concrete insulation layer subgrade, when the insulation layer thickness exceeds 0.3m, the influence of foam concrete insulation layer on the freezing depth and frost heaving amount of subgrade is weak. Due to the addition of XPS insulation board on the shady slope shoulder and sunny slope shoulder, the cold insulation effect is enhanced. When the thickness of insulation layer exceeds 0.3m, the anti-frost heave relief of subgrade is still obvious. The composite structure insulation layer can weaken sunny-shady slopes effect of the subgrade in the seasonal frozen soil area. The subgrade transverse differential frost heave deformation is suppressed. When the thickness of insulation layer increases to 0.4m, the maximum freezing depth and maximum frost heave amount of on the shady slope shoulder and sunny slope shoulder decrease to 1.09m, 0.84m, 9mm and 6mm respectively. When the thickness of insulation layer is greater than 0.2m, the anti-frost heave effect of composite insulation structure tends to decline steadily. Therefore, when the foamed concrete insulation layer is used as the anti-frost heave measure for the subgrade in the seasonal frozen soil area, the 0.3m thick foamed concrete insulation layer is selected as the best measure to inhibit frost heave development for the subgrade section without significant sunny-shady slopes effect. For the subgrade section with significant shady and sunny slope effect, the composite insulation structure measures of over 0.2m are selected. This can inhibit the development of frost heave of the whole subgrade and maintain the thermal stability balance of the subgrade.

**Table 4:** The maximum freezing depth of thermal insulation layer subgrade and composite thermal insulation structure subgrade.

Maximum freezing depth/ m Insulation layer thickness/ m	Thermal insulation layer subgrade/ m			Composite thermal insulation structure subgrade/ m		
	Shaded slope shoulder	Subgrade center	Sunny slope shoulder	Shaded slope shoulder	Subgrade center	Sunny slope shoulder
0.0	2.40	1.80	2.01	2.40	1.80	2.01
0.1	2.13	1.35	1.72	1.62	1.31	1.45
0.2	1.96	1.02	1.53	1.40	1.00	1.21
0.3	1.85	0.70	1.35	1.23	0.60	1.01
0.4	1.82	0.67	1.32	1.09	0.52	0.84

**Table 5:** The maximum frost heave deformation of thermal insulation layer subgrade and composite thermal insulation structure subgrade.

Maximum frost heave deformation/ mm Insulation layer thickness/ m	Thermal insulation layer subgrade/ mm			Composite thermal insulation structure subgrade/ mm		
	Shaded slope shoulder	Subgrade center	Sunny slope shoulder	Shaded slope shoulder	Subgrade center	Sunny slope shoulder
0.0	27	18	22	27	18	22
0.1	21	12	17	14	10	11
0.2	19	10	15	12	9	10
0.3	16	6	13	11	5	9
0.4	15	3	12	9	2	6

## 4. Conclusion

In this manuscript, the hydrothermal multi-field coupling simulation is carried out for the subgrade of foamed concrete insulation layer and the composite insulation structure (XPS insulation board + foamed concrete). The influence of different insulation measures on frost heave deformation characteristics of subgrade is analyzed. The freezing depth and maximum frost heave under different insulation thickness of subgrade center, shady slope shoulder and sunny slope shoulder are discussed. The following conclusions are obtained:

1. From the perspective of hydrothermal deformation of subgrade, the variation laws of temperature, maximum freezing depth, ice content and maximum frost heave of subgrade with different anti-frost heave measures are analyzed. The influence of foamed concrete insulation thickness on the characteristics of frost heave of subgrade is discussed.
2. The temperature, freezing depth, ice content and maximum frost heaving amount between common subgrade and insulation subgrade are compared. The simulation results show that the maximum freezing depth of common subgrade center in the seasonal frozen soil area in the study area is 1.8m. The maximum frost heaving amount is 18mm. No matter the foamed concrete insulation layer or the composite structure insulation layer, the maximum freezing depth of the subgrade can be less than 1.0m, and the maximum frost heave volume can be less than 10mm, when the insulation layer thickness exceeds 0.2m.
3. The heat budget of subgrade soils does not change with the increase of insulation layer thickness. For the subgrade section without significant shady and sunny slope effect, foamed concrete insulation layer should be used, and the thickness of insulation layer should be 0.3m. For the subgrade section with significant shady and sunny slope effect, the composite insulation structure (XPS insulation board + foamed concrete) should be adopted. The thickness of the insulation layer should be greater than 0.2m. It is helpful to restrain the uneven frost heave deformation of subgrade in seasonal frozen soils areas. It is conducive to improving the overall stability and long-term service performance of subgrade.

## Supporting information

S1 Data (XLSX)
